# *Candida albicans* Mycofilms Support *Staphylococcus aureus* Colonization and Enhances Miconazole Resistance in Dual-Species Interactions

**DOI:** 10.3389/fmicb.2017.00258

**Published:** 2017-02-23

**Authors:** Ryan Kean, Ranjith Rajendran, Jennifer Haggarty, Eleanor M. Townsend, Bryn Short, Karl E. Burgess, Sue Lang, Owain Millington, William G. Mackay, Craig Williams, Gordon Ramage

**Affiliations:** ^1^Oral Sciences Research Group, Glasgow Dental School – School of Medicine, Dentistry and Nursing, College of Medical, Veterinary and Life Sciences, University of GlasgowGlasgow, UK; ^2^Institute of Healthcare Policy and Practise – Institute of Healthcare Associated Infection, University of the West of ScotlandPaisley, UK; ^3^Glasgow Polyomics, University of GlasgowGlasgow, UK; ^4^Department of Life Sciences, School of Health and Life Sciences, Glasgow Caledonian UniversityGlasgow, UK; ^5^Strathclyde Institute of Pharmacy and Biomedical Sciences, University of StrathclydeGlasgow, UK

**Keywords:** Candida albicans, Staphylococcus aureus, miconazole, biofilm, extracellular DNA

## Abstract

Polymicrobial inter-kingdom biofilm infections represent a clinical management conundrum. The presence of co-isolation of bacteria and fungi complicates the ability to routinely administer single antimicrobial regimens, and synergy between the microorganisms influences infection severity. We therefore investigated the nosocomial pathogens *Staphylococcus aureus* and *Candida albicans* with respect to antimicrobial intervention. We characterized the interaction using biofilm assays and evaluated the effect of miconazole treatment using *in vitro* and *in vivo* assays. Finally, we assessed the impact of biofilm extracellular matrix (ECM) on these interactions. Data indicated that the *C. albicans* mycofilms supported adhesion and colonization by *S. aureus* through close interactions with hyphal elements, significantly increasing *S. aureus* biofilm formation throughout biofilm maturation. Miconazole sensitivity was shown to be reduced in both mono- and dual-species biofilms compared to planktonic cells. Within a three-dimensional biofilm model sensitivity was also hindered. *Galleria mellonella* survival analysis showed both enhanced pathogenicity of the dual-species infection, which was concomitantly desensitized to miconazole treatment. Analysis of the ECM revealed the importance of extracellular DNA, which supported the adhesion of *S. aureus* and the development of the dual-species biofilm structures. Collectively, these data highlight the clinical importance of dual-species inter-kingdom biofilm infections, though also provides translational opportunities to manage them more effectively.

## Introduction

Despite their clinical significance, polymicrobial biofilm infections continue to be a widely understudied health problem. Advancement in biofilm research has highlighted that these cellular communities are rarely composed of a single-species consortia, but instead exist as complex, diverse, and heterogeneous structures (Peleg et al., 2010). By actively participating within these environments different micro-organisms can interact at a mechanistic level through direct and indirect exchanges of both a physical and chemical nature. This can ultimately influence disease severity by promoting intensified pathogenic phenotypes (Peters et al., 2010), including increased recalcitrance to both host defenses and antimicrobial therapies (Kart et al., 2014). Although belonging to distinct phylogenetic kingdoms, the dimorphic fungus *Candida albicans* and the bacterial pathogen *Staphylococcus aureus* possess the ability to habitually co-exist as complex polymicrobial biofilms within the human host, and are therefore key exemplars of polymicrobiality.

*Staphylococcus aureus* and *C. albicans* are defined as the second and fourth most commonly cultured organisms from bloodstream infections within the USA (Wisplinghoff et al., 2004). An estimated 27% of candidaemia infections are polymicrobial, with *S. aureus* representing the third most frequently co-isolated organism (Klotz et al., 2007). Duality of infection is also frequently observed in co-infections of burn wounds and cystic fibrosis, as well from the surface of indwelling medical devices such as dentures, prosthetic joints and implants, and more commonly catheters (Valenza et al., 2008; Peters et al., 2012a). Mechanistically, there is evidence to suggest that this interaction is multifaceted, intricately linked to biofilm-phased development. Initial attachment of *S. aureus* to C. *albicans* hyphae appears to be mediated by *C. albicans* agglutinin like sequence 3 protein (Als3p; Peters et al., 2012b). As the biofilm develops, quorum sensing (QS) molecules have been shown to exhibit both synergistic and antagonistic effects, with either organism secreting reciprocal QS molecules to positively and negatively influence biofilm growth (Jabra-Rizk et al., 2006; Lin et al., 2013). Structurally, the extracellular matrix (ECM) of *C. albicans* has been shown to advantageously support these dual-species biofilms through prevention of diffusion of vancomycin to access *S. aureus*, with the bacteria potentially encasing itself within this polymeric material (Harriott and Noverr, 2009). Furthermore, it has now been shown that the fungal specific matrix component β-1,3-glucan promotes this vancomycin resistance (Kong et al., 2016). However, how these biofilms respond to antifungal agents remains undefined.

*Candida albicans* biofilms are notoriously resistant to antifungal agents, displaying up to 1000-fold increases in resistance to azoles compared to their planktonic counterparts (Ramage et al., 2001). Triazoles, such as fluconazole, represent the first-line option for *C. albicans* infections, though are often ineffectual against *C. albicans* biofilms (Sherry et al., 2014; Rajendran et al., 2016b). However, unlike these more commonly used triazoles, the imidazole miconazole appears to display fungicidal activity against *C. albicans* biofilms *in vitro* (Vandenbosch et al., 2010). This same agent has also been shown to display antibacterial activity against a range of Gram-positive bacteria, including *S. aureus* (Nobre et al., 2010). We therefore sought to evaluate the *in vitro* and *in vivo* efficacy of miconazole against *C. albicans* and *S. aureus* dual-species biofilms, and aimed to understand the contribution of the ECM in this interaction. Here we report that *C. albicans* mycofilms actively enhance *S. aureus* colonization and negatively impacts miconazole sensitivity.

## Materials and Methods

### Microbial Growth and Standardization

*Candida albicans* SC5314 strain and the biofilm defective *Staphylococcus aureus* Newman strain were used throughout the duration of this study (Duthie and Lorenz, 1952; Fonzi and Irwin, 1993; Beenken et al., 2003). *C. albicans* SC5314 was grown and maintained on Sabouraud’s dextrose agar (Oxoid, Hampshire, UK) and incubated at 30°C for 48 h. Yeast cells were propagated by inoculating yeast peptone dextrose (YPD) media (Sigma–Aldrich, Dorset, UK) with a loopful of colonies and incubated at 30°C at 200 rpm in an orbital shaker overnight. After growth, cells were pelleted by centrifugation and washed in phosphate buffered saline [PBS (Sigma–Aldrich, Dorset, UK)] before being standardized in selected media at assay specific concentration requirements after cells were counted using a haemocytometer. The *S. aureus* Newman strain was cultured and maintained on Luria Bertani agar at 37°C for 48 h. *S. aureus* cells were propagated overnight in Luria Bertani broth at 37°C before being washed by centrifugation in PBS. Following cell washing, they were standardized in selected media to assay specific concentration requirements after their density was determined using a colorimeter.

### Development of Dual-Species Biofilm Model

Both *C. albicans* and *S. aureus* were standardized to 1 × 10^6^ cells/mL in 50% v/v foetal bovine serum (FBS) diluted in sterile water. Biofilms were formed at 37°C over 1.5 (90 min), 6 and 24 h by adding both organisms 1:1 to either microtiter plates (Corning, NY, USA) or 13 mm diameter Thermanox^TM^ coverslips (Fisher Scientific, Loughborough, UK), depending on the assay function. Mono-species biofilms and appropriate media controls were run in parallel for each experimental condition assessed. Following incubation media was discarded and biofilms were washed with PBS to remove any non-adherent cells. The biomass of each biofilm was quantified by the crystal violet (CV) assay described previously (Sherry et al., 2014). The CV absorbance was then measured spectrophotometrically at a wavelength of 570 nm using a microtiter plate reader (FLUOStar Omega, BMG Labtech, Aylesbury, UK).

### Biofilm Composition and Viability Analysis

Biofilm composition was enumerated using live/dead PCR, a technique also able to differentiate between total and live cells using methodologies previously described by our group (Townsend et al., 2016). Biofilms were grown as above on Thermanox^TM^ coverslips (Fisher Scientific, Loughborough, UK), washed in PBS, and sonicated in 1 mL of PBS at 35 kHz in an ultrasonic water-bath (Fisher Scientific, Loughborough, UK) for 15 min to remove the biofilm. Next, 50 μM of propidium monoazide [PMA (Cambridge Bioscience, Cambridge, UK)] was added to the sample and incubated for 10 min in the dark to allow uptake of the dye. PMA-free controls were also included to determine total cell number. All samples were then exposed to a 650 W halogen light for 5 min before DNA was extracted using the QIAamp DNA mini kit, as per manufacturer’s protocol (Qiagen, Crawley, UK). Real-time quantitative PCR (qPCR) was then used to determine the live and total cell number within each biofilm. Briefly, 1 μL of extracted DNA was added to a PCR mastermix containing Fast SYBR^®^ Green Master Mix, 10 μM species-specific forward and reverse primers (**Table 1**), and RNase free water. qPCR was then carried out using the Step-One plus real time PCR machine (Life Technologies, Paisley, UK) using the following thermal profile: 50°C for 2 min, 95°C for 2 min, followed by 40 cycles of 95°C for 3 s and 60°C for 30 s. Colony forming equivalents (CFE) were calculated compared to a standard curve of serially diluted DNA of each species as previously described (O’Donnell et al., 2016).

**Table 1:** Primer sequences used for qPCR.

Primer	Sequence (5′-3′)	Reference
*Candida albicans*	F – GAGCGTCGTTTCTCCCTCAAACCGCTGG R – GGTGGACGTTACCGCCGCAAGCAATGTT	Alves et al., 2014
*Staphylococcus aureus*	F – ATTTGGTCCCAGTGGTGTGGGTAT R – GCTGTGACAATTGCCGTTTGTCGT	O’Donnell et al., 2016
18S	F – CTCGTAGTTGAACCTTGGGC R – GGCCTGCTTTGAACACTCTA	Sherry et al., 2016
16S	F – CGCTAGTAATCGTGGATCAGAATG R – TGTGACGGGCGGTGTGTA	Sherry et al., 2016

### Dual-Species Biofilm Visualization

Mono- and dual-species biofilms were stained with 5 μM calcofluor white (Invitrogen, Paisley, UK) and 20 μM SYTO9^®^ (Sigma–Aldrich, Dorset, UK) was used to stain both fungal and bacterial cells. Biofilms were then imaged using a confocal laser scanning microscopy (CLSM) microscope (Leica SP5 laser scanning confocal microscope) at an excitation and emission wavelength of 350 and 400 nm for calcofluor white and 485 and 500 nm for SYTO9^®^. Images were then processed and analyzed using Volocity 3D Image Analysis Software (Perkin Elmer). In addition, biofilms were also imaged using scanning electron microscopy (SEM). After biofilm development, biofilms were fixed using 2% para-formaldehyde, 2% glutaraldehyde, 0.15 M sodium cacodylate, and 0.15% w/v alcian blue and processed for SEM, as previously described (Erlandsen et al., 2004). Samples were then sputter coated in gold before being imaged using a JEOL JSM-6400 scanning electron microscope.

### *In vitro* Biofilm Susceptibility Testing

Simple mono- and dual-species biofilms were grown in microtiter wells for 24 h before being challenged for a subsequent 24 h with serially twofold diluted concentrations of miconazole (Sigma–Aldrich, Dorset, UK), ranging from 320 to 0.63 mg/L using standardized biofilm testing methodology to determine the sessile minimum inhibitory concentration (SMIC; Sherry et al., 2014). Planktonic MIC (PMIC) testing was also performed on single- and dual-species inoculum using standard CLSI broth microdilution testing (CLSI, 2012). In addition, these biofilms were also treated with miconazole ± DNase I [130 mg/L (Sigma–Aldrich, Dorset, UK)], lyticase, and chitinase [50 mg/L (Sigma–Aldrich, Dorset, UK)]. Following treatment the proportional viability was calculated in each test condition compared to an untreated control using the XTT metabolic reduction assay (Pierce et al., 2008).

In parallel, we prepared complex three-dimensional mono- and dual-species biofilms for 24 h on a 3D cellulose matrix model recently optimized and described by our group (Townsend et al., 2016). After growth, biofilms were gently washed with PBS before being treated with 40 mg/L of miconazole for a further 24 h. Following treatment, biofilms were washed before being sonicated for 5 min in 1 mL of PBS at 35 kHz in an ultrasonic water-bath (Fisher Scientific, Leicestershire, UK) to remove biofilm cells, and the viability of the biofilms after treatment was determined using species-specific live/dead PCR, as described above.

### *Galleria mellonella* Survival Assays

The pathogenicity of *C. albicans, S. aureus*, and co-infection was assessed using the *Galleria mellonella* killing assay as previously described (Rajendran et al., 2015). Sixth instar *G. mellonella* larvae (Livefoods Direct Ltd, UK) with a body weight between 200 and 300 mg were used. For each group, 30 larvae were injected directly into the hemocoel at a concentration of 5 × 10^5^ CFU/larvae/organism (1 × 10^6^ CFU/larvae total for co-infection) through the hindmost right proleg region, using a 50 μL Hamilton syringe with a 26 g needle. Larvae were then placed in Petri dishes at 37°C, with survival being monitored across a 48 h period. Larvae were considered dead when they displayed no movement in response to touch and showed severe melanisation within the cuticle as previously described (Sherry et al., 2014). To determine fungal and bacterial burden after 24 h post-infection, three representative larvae from each group were homogenized and DNA extracted as described elsewhere (Rajendran et al., 2015). The fungal and bacterial burden, expressed as CFE (CFE/100 mg body weight), was then assessed using species-specific primers and standard curves as described above. For antimicrobial treatment assays, larvae were administered with miconazole in the hindmost left proleg region 2 h post-infection of either mono- or dual-species co-infection. A miconazole concentration of 75 mg/kg body weight was used throughout this experiment. Appropriate uninfected, drug only and vehicle controls were included for each experiment.

### Quantification of eDNA Release

The quantity of eDNA released into the biofilm supernatant and ECM from *C. albicans, S. aureus*, and dual-species biofilms was measured using a microplate fluorescence assay (MFA), as previously described (Rajendran et al., 2014). Standardized cells (1 × 10^6^ cells/mL) were seeded in a black 96-well microtiter plate (Corning, NY, USA) and SYBR^®^ Green I (Invitrogen, Paisley, UK) added to the cells at a ratio of 1:10. The binding of this dye results in fluorescence that is directly proportional to the level of eDNA. These levels were then measured after 6, 12, and 24 h of growth using a microtiter plate reader at excitation and emission wavelengths of 485 and 518 nm, and eDNA quantified in comparison to a standard curve. Next, in order to determine the quantity of eDNA contributing to the ECM the biomass of 24 h grown biofilms was removed using a cell scraper and then treated with 0.2 M EDTA to extract the ECM. Samples were then centrifuged at 10,000 × *g* and EDTA supernatants recovered. The quantity of ECM associated eDNA was then calculated using the MFA described above. In addition, DNA was precipitated from the isolated matrix using ammonium acetate precipitation and species-specific eDNA was quantified using qPCR as described above, using fungal specific 18S and bacteria specific 16S primers (**Table 1**).

### *Staphylococcus aureus* Mycofilm Adherence Assay

For adhesion, 24 h grown *C. albicans* biofilms were treated ± DNase I [130 and 650 mg/L (Sigma–Aldrich, Dorset, UK)] prepared in a buffer solution containing 0.15 M NaCl with 5 mM MgCl_2_ for 4 h at 37°C. *S. aureus* cells were standardized to 1 × 10^6^ CFU/mL before being stained with 20 μM SYTO9^®^ for 20 min, prior to excess dye being removed by centrifugation. Biofilms were then washed again before the addition of the fluorescently stained *S. aureus* cells to the *C. albicans* biofilm for 90 min. After incubation, biofilms were washed again to remove any non-adherent *S. aureus* cells and SYTO9^®^ fluorescence quantified using plate reader at an excitation and emission wavelengths of 485 and 518 nm, respectively. The number of adherent cells was then quantified in comparison to a serially diluted *S. aureus* standard curve.

### Statistical Analysis

Graph production, data distribution, and statistical analysis were performed using GraphPad Prism (version 5; La Jolla, CA, USA). Student *t*-tests were used to analyze experiments comparing independent sample data. Kaplan Meier survival curves were analyzed using the log-rank test. Statistical significance was achieved if *P <* 0.05.

## Results

### *Staphylococcus aureus* Utilizes *Candida albicans* Mycofilms as a Structural Scaffold

**FIGURE 1:**
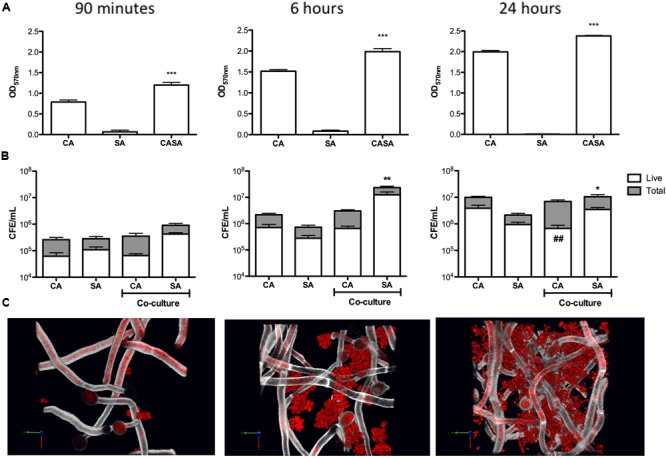
***Candida albicans* mycofilms facilitates *Staphylococcus aureus* biofilm formation.** Mono- and dual-species biofilms were standardized 1 × 10^6^ CFU/mL and grown on 96-well plates for 90 min, 6 and 24 h. Biofilms were then washed with phosphate buffered saline (PBS) and biomass assessed using the crystal violet assay **(A).** Standardized biofilms were grown before being sonicated to remove the biomass. Live/dead PCR was then used to extract DNA and determine total and live colony forming equivalents (CFE) from mono- and dual-species biofilms **(B)**. Biofilm morphology was then analyzed using CSLM. Biofilms were grown before being fluorescently stained using calcofluor white and SYTO9^®^ dyes. Resulting biofilms were then viewed on a Leica SP5 laser scanning confocal microscope and images were then processed and analyzed using Volocity 3D Image Analysis Software **(C)**. Results represent data from three independent occasions. Statistical analysis compares dual-species biofilms to their mono-species equivalent (^∗^*p* < 0.05, ^∗∗^*p* < 0.01, ^∗∗∗^*p* < 0.001). ^##^*p* < 0.01, compares dead cells between dual- and mono-species biofilms.

First, we wanted to determine the basis of how *C. albicans* and *S. aureus* interact within a biofilm environment. We purposely used the Newman’s strain of *S. aureus* that has a defective biofilm phenotype (Beenken et al., 2003; Supplementary Figure 1), working with the hypothesis that *C. albicans* biofilm structure positively influenced its ability to colonize and form biofilms irrespective of host derived substrates. Mono- and dual-species biofilms were quantified at early (90 min), intermediate (6 h), and mature (24 h) stages of biofilm growth using the CV biomass assay (**Figure 1A**). It was shown that dual-species biofilms contained significantly more biomass than either of the mono-species organism’s biofilms at 90 min, 6 and 24 h (*p* < 0.001). A sensitive quantitative molecular approach was then used in order to assess both viability and composition of each species within the dual-species biofilm throughout maturation. After 90 min no statistical differences were observed in the overall total and viable number of *C. albicans* between the mono- and dual-species biofilms (*p* = 0.1021), though a 2.76- and 3.92-fold increase in the total and live number of *S. aureus* cells was observed within the dual-species biofilm, respectively (**Figure 1B**). When scrutinized visually we observe *C. albicans* cells existing as germ tubes with single and small clusters of S. *aureus* cells adhered to these structures (**Figure 1C**). As the biofilm progressed to the intermediate phase of growth at 6 h no difference was observed in the total and live number of *C. albicans* cells between mono- and dual-species biofilm cultures (**Figure 1B**). Though, a significant *S. aureus* increase within the dual-species biofilm was observed (*p* < 0.01, 24.11-fold), which is reinforced microscopically where we observe enhanced colonies of clustered *S. aureus* cells attached to the hyphae of *C. albicans* (**Figure 1C**). Next, when we evaluated fully matured 24 h biofilms we observe similar trends as above (**Figure 1B**), with no statistical difference between the total numbers of *C. albicans* cells between both biofilms. Intriguingly though, when we quantified the number of dead cells (total cells minus live cells) we observed a statistically significant increase in the quantity of dead *C. albicans* cells in the dual-species biofilm (*p* < 0.01, 2.52-fold). Conversely, *S. aureus* was significantly increased in the dual-species biofilm compared to mono-species biofilms, with total and viable cells number increasing 5.85- and 3.66-fold, respectively (*p* < 0.05). Visually, these dual-species biofilms were characterized by extensively spread micro-colonies of *S. aureus* biofilm cells attached to and interspersed throughout the dense network of *C. albicans* hyphal growth (**Figure 1C**). Collectively, these data suggest that these two microorganisms display a level of synergy given their intimate physical relationship, where candidal mycofilms serve as the base substrate for *S. aureus* colonization.

### Dual-Species Biofilms Decrease Miconazole Efficacy *In vitro*

First, we investigated the sensitivity of simple mono- and dual-species planktonic and sessile cells using standardized metabolic based methodologies. It was shown that the PMIC for *S. aureus* was 1 mg/L and *C. albicans* and dual-species were 4 mg/L. The SMIC_80_, i.e., the concentration that inhibited 80% metabolic activity of the biofilm, was 10-fold greater for each biofilm tested, with *S. aureus* mono-species biofilms at 10 mg/L, whereas for both the *C. albicans* mono-species and the dual-species biofilms at 40 mg/L (**Table 2**).

**Table 2:** Sessile MIC for miconazole in the presence and absence of hydrolytic enzymes.

Minimum inhibitory concentration (mg/L)				
	**SMIC_80_**			
	**Miconazole**	**Miconazole + DNase**	**Miconazole + Lyticase**	**Miconazole + Chitinase**
*C. albicans* (CA)	40	40	40	40
*S. aureus* (SA)	10	10	10	10
CA+SA	40	20	40	40

**FIGURE 2:**
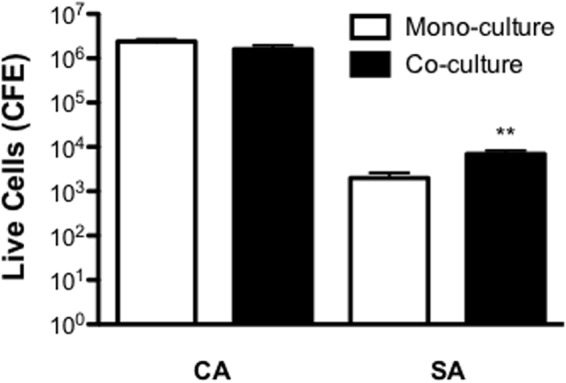
**Inter-kingdom interactions decrease *Staphylococcus aureus* sensitivity to miconazole.** Biofilms were grown for 24 h on a cellulose matrix based hydrogel model before being washed with PBS and treated with 40 mg/L of miconazole for a further 24 h. After treatment, the cellulose matrix was removed and sonicated to dislodge the biofilm biomass. Live/dead PCR was then used to extract DNA and quantify total and live CFE. Data is presented as the CFE of live cells comparing treated mono- and dual-species biofilms. Data represents duplicate samples from three independent time points with significance achieved with ^∗∗^*p* < 0.01.

Next, we evaluated mono- and dual- species biofilms within a more complex 3D wound model determined their sensitivity against a 24 h treatment of miconazole (SMIC_80_ = 40 mg/L). No significant differences were observed for *C. albicans* post-treatment between mono- and dual-species biofilms (**Figure 2**). However, there was decreased sensitivity observed in *S. aureus* when grown in the dual-species biofilm, with a significant 3.52-fold increase in the quantity of live cells post-treatment (*p* < 0.01; **Figure 2**).

### Dual-Species Biofilms Decrease Miconazole Efficacy *In vivo*

**FIGURE 3:**
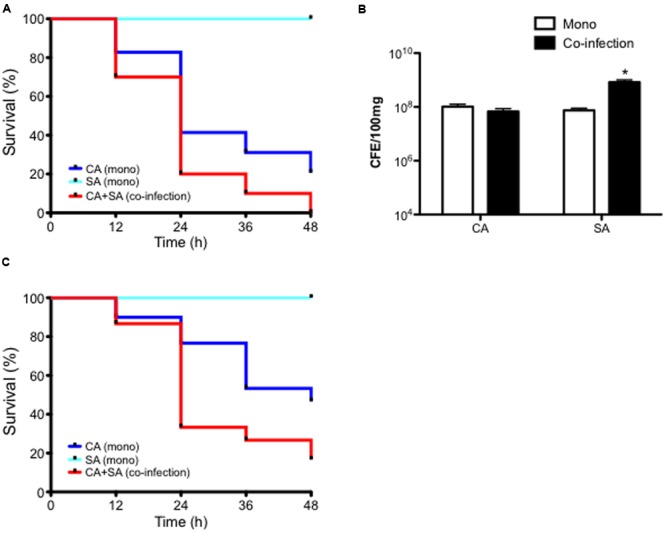
***Candida albicans* and *Staphylococcus aureus* display synergistic pathogenicity and reduced miconazole sensitivity *in vivo*.** *C. albicans* (CA) and *S. aureus* (SA) were standardized to 5 × 10^5^ CFU/larvae and administered to larvae as a mono or co-infection (CA+SA) with percentage survival monitored across a 48 h period and data presented using a Kaplan Meier plot. Data are derived from three independent groups of 10 larvae with significance (^∗^*p* < 0.05, ^∗∗^*p* < 0.001) determined using the log-rank test in comparison between *C. albicans* and *S. aureus* alone and co-infection **(A)**. After 24 h post-infection, representative larvae were snap frozen in liquid nitrogen and DNA extracted. Microbial burden was then determined using qPCR and presented as CFE **(B)**. Data are derived from three larvae with ^∗^*p* < 0.05. Upon 2 h post-infection of larvae with 5 × 10^5^ CFU/larvae with CA, SA, and co-infection (CA+SA), larvae were administered with 75 mg/kg of miconazole. Percentage survival was monitored across a 48 h period and represented with a Kaplan Meier plot **(C)**. Data represents results from three independent groups of 10 larvae with significance achieved (^∗∗^*p* < 0.01, ^∗∗∗^*p* < 0.001) comparing *C. albicans, S. aureus*, and co-infection using the log-rank test.

In order to first establish whether any co-operative pathogenicity exists between both organisms *in vivo*, a *G. mellonella* infection model was utilized. Inoculation of larvae with *C. albicans* was shown to kill approximately 60 and 80% of larvae after 24 and 48 h post-infection, respectively. Conversely, larvae co-infected with both *C. albicans* and *S. aureus* demonstrated an 80 and 100% mortality rate after 24 and 48 h, respectively. This was a significant increase compared to both *C. albicans* (*p* < 0.05) only and *S. aureus* only (*p* < 0.001), the latter of which was avirulent at the chosen cell concentration (**Figure 3A** and Supplementary Figure 2). Three selected larvae were then chosen to determine the microbial burden at 24 h post-infection. Results showed that *C. albicans* CFE counts remained at equivalent levels in mono- and dual-infection. Whereas, as observed with the *in vitro* data the quantity of *S. aureus* was significantly enhanced 11.1-fold when co-infected with *C. albicans* (*p* < 0.05; **Figure 3B**). These data indicate that when in combination the pathogenic outcome is synergised in co-infection, and given that *S. aureus* is quantitatively enhanced then this would suggest *C. albicans* supports and enhances its growth.

Next, we tested the efficacy of miconazole against this synergised co-infection using the *G. mellonella* infection model. Upon 2 h post-infection larvae were administered with miconazole and mortality monitored over a 48 h period. Results showed that after 24 h, over 75% of *C. albicans* infected larvae remained alive after miconazole treatment, and almost 50% survived 48 h after infection. All larvae remained alive at 48 h post-inoculation with *S. aureus*. However, co-infection with both organisms resulted in a loss of susceptibility to miconazole, with mortality reaching almost 70% after 24 h and >85% at 48 h post-infection, a significant difference in larvae survival compared to *C. albicans* (*p* < 0.01) and *S. aureus* (*p* < 0.001) alone (**Figure 3C**).

### Extracellular DNA Promotes Stability to the ECM of Dual-Species Biofilms

**FIGURE 4:**
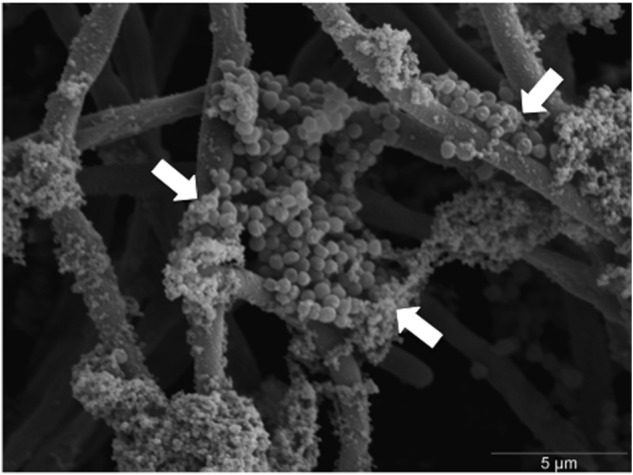
**Scanning electron micrograph of *S. aureus* colonizing *C. albicans* hyphae within dual-species biofilms.** Dual-species biofilms were grown for 24 h before being fixed, processed, and imaged using a JEOL-JSM 6400 scanning electron microscope. *S. aureus* colonies can be seen adhering and embedded within the hyphal meshwork of *C. albicans*. White arrows indicate clusters of *S. aureus* colonies encased within extracellular matrix (ECM). Scale bar represents 5 μm at × 5000 magnification.

When analyzed visually through SEM, clusters of *S. aureus* biofilm can be seen embedded within *C. albicans* mycofilms and associated with ECM (**Figure 4**). Given that ECM is an important characteristic associated with antifungal resistance, we hypothesized that degradation of ECM components in combination with miconazole would enhance biofilm sensitivity. It was shown that degradation of eDNA, β-1,3-glucan and chitin had no effect on the MIC_80_ value for *C. albicans* and *S. aureus* mono-species biofilms. However, when these same components were degraded by DNase in the dual-species biofilm, we observed a twofold increase in miconazole sensitivity, but no change when treated in combination with the other enzymes (**Table 2**).

**FIGURE 5:**
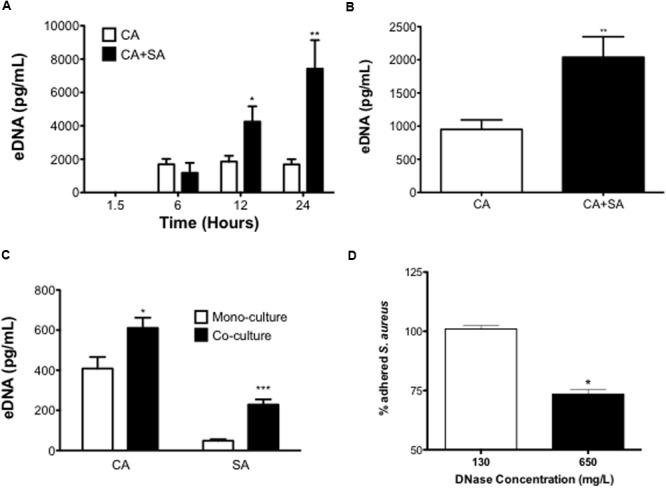
**Extracellular DNA contributes to inter-kingdom pathogenicity.** Mono- and dual-species biofilms were seeded at 1 × 10^6^ CFU/mL in black 96-well plates and eDNA release at 1.5, 6, 12, and 24 h measured using a SYBR^®^ Green 1 based microplate fluorescence assay (MFA) in comparison to a standard curve **(A)**. Biofilms were washed with 0.2 M EDTA to remove the ECM and resulting eDNA quantified using the MFA described above in comparison to a standard curve **(B)**. ECM associated DNA was then precipitated from matrix extracts and species contributions were analyzed using qPCR **(C)**. *C. albicans* only biofilms were grown for 24 h in black 96-well plates. After washing biofilms were then treated with either 130 or 650 mg/L of DNase for 4 h. SYTO9^®^ stained *S. aureus* cells (1 × 10^6^ CFU/mL) were then added to the biofilm and incubated for 90 mins before being fluorescently quantified in comparison to an vehicle control treated biofilm **(D).** Data represents duplicate samples from three independent experiments (^∗^*p* < 0.05, ^∗∗^*p* < 0.01, ^∗∗∗^*p* < 0.001).

To determine whether *C. albicans* positively influenced this dual-species interaction we next investigated the mechanistic contribution of the ECM, hypothesizing that eDNA may be pivotal factor in supporting the dual-species interactions, as has been shown in mono-species *C. albicans* biofilms (Rajendran et al., 2014). **Figure 5A** shows an increase of eDNA release into the biofilm supernatant from dual-species biofilm compared to a *C. albicans* biofilm in a time dependent manner. Throughout the assessed time points, mono-species *S. aureus* biofilms eDNA levels were below detectable limit. After 90 min of biofilm development eDNA levels of all tested groups remained below a detectable level. After 6 h of growth, there was no significant difference between *C. albicans* and dual-species eDNA release (*p* = 0.4296). At 12 h, a significant increase in the quantity of eDNA released from the dual-species biofilm was observed (2.28-fold, *p* < 0.05). After 24 h the quantity of eDNA released from *C. albicans* biofilms plateaued, whereas the dual-species biofilm continued to rise, with a significant increase (*p* < 0.01, 4.40-fold).

Finally, we next assessed if this secreted eDNA contributed to the biofilm structure by measuring the eDNA quantity within the ECM. It was shown that the ECM associated eDNA significantly increased 2.15-fold when comparing the dual-species biofilm to the *C. albicans* biofilm (*P* < 0.01; **Figure 5B**). We then used qPCR to analyze the species contribution to this increase, showing that in the dual-species biofilm both organisms released significantly more eDNA than their mono-species counterparts. The quantity of *C. albicans* eDNA significantly increased from 408.5 to 611.1 pg/mL in the co-culture (*p* < 0.05), with the *S. aureus* eDNA also significantly increasing 4.7-fold (*p* < 0.001; **Figure 5C**). Due to the increase of eDNA in the dual-species biofilm we then investigated its role in supporting the adhesion of *S. aureus* to a mature *C. albicans* biofilm. It was shown that a 4 h DNase treatment at 130 μg/mL did not have any detectable effect on *S. aureus* adhesion compared to the control, whereas when the DNase concentration was increased (650 μg/mL) we observed a significant 26.5% reduction in the number of *S. aureus* cells that adhered to the *C. albicans* biofilm (*P* < 0.05; **Figure 5D**).

## Discussion

Polymicrobial infections are increasingly recognized as a clinically important entities due to altered patient prognosis that results in increased hospital stays and decreased antimicrobial efficacy (Sancho et al., 2012). In this manuscript, we aimed to characterize the inter-kingdom interactions between the two most common nosocomial pathogens *C. albicans* and *S. aureus*, and subsequently evaluate miconazole *in vitro* and *in vivo* sensitivities, an antifungal with reported cross-kingdom efficacy. Using this strategy it was shown that *S. aureus* is able to use *C. albicans* as a physical scaffold to colonize and form biofilms upon an existing biofilm, a phenomenon we have termed as mycofilms. We also report a novel role for eDNA in promoting the stability of *C. albicans*/*S. aureus* biofilms that augments biofilm mediated resistance to miconazole.

An initial objective from this series of studies was to create a reliable functional assay to quantify and characterize the interaction between *C. albicans* and *S. aureus.* It was our hypothesis based on previous studies that *C. albicans* hyphae are integral to supporting *S. aureus* biofilm formation (Peters et al., 2012b). To test this hypothesis we used the biofilm defective *S. aureus* Newman strain to determine whether the presence of *C. albicans* could positively enhance its ability to colonize and form biofilms. Indeed, our hypothesis was confirmed with the observation that single *S. aureus* cells and small clusters adhere to *C. albicans* germ tubes after only 90 min of growth, while synchronously enhancing overall biomass, significantly increasing throughout biofilm maturation. This is in line with previous reports predicting the initiation of polymicrobial biofilm occurs upon initial *C. albicans* germ tube formation (Harriott and Noverr, 2009), and continues as the biofilm becomes fully mature (Peters et al., 2010). Given that the ALS3 gene is highly expressed during early phases of *C. albicans* filamentous growth during biofilm formation (Sherry et al., 2014), then it is unsurprising that this key biofilm adhesin is involved in mediating the initial attachment of bacteria within the polymicrobial biofilm (Peters et al., 2012b). Als3p shares between 80 and ≥95% homology with collagen binding and clumping factor proteins of *S. aureus* (Sheppard et al., 2004), therefore the cellular basis for these interactions may be reciprocal. Indeed, the anti-candidal vaccine derived from the Als3p also protects against *S. aureus* infection in a murine model (Spellberg et al., 2008). Interestingly, the use of the biofilm defective Newman’s strain of *S. aureus* suggests that this species does not necessarily require the full armamentarium of biofilm-associated genes, as the presence of a scaffold of *C. albicans* hyphae is capable of facilitating the process, which may explain why these organisms as so frequently co-isolated (Beenken et al., 2003). This may also explain why there is such heterogeneity in terms of *S. aureus* biofilm formation (Smith et al., 2008), a phenomenon also evident in *C. albicans* biofilm formation (Sherry et al., 2014; Rajendran et al., 2016a).

As *C. albicans* biofilm maturation progressed the quantities of *S. aureus* was significantly enhanced, though notably there was a significant increase in the number of dead *C. albicans* cells, suggesting that *S. aureus* is able to utilize *C. albicans* in some undefined way through this close interaction. Metabolomic analysis of the interaction enabled us to recently identify that *S. aureus* is able to consume sterols, products also found in the secretome of *C. albicans* biofilms (Weidj et al., 2016). Given that sterols have been shown to promote *S. aureus* growth (Shine et al., 1993), then this suggests that sterol consumption is a contributing factor to its increased growth rates in the dual-species biofilm. Other metabolites, such as the *C. albicans* secreted QS compound farnesol has also been shown to modulate *S. aureus* behavior through inhibiting lipase activity and biofilm formation, as well as improving its sensitivity to gentamicin (Jabra-Rizk et al., 2006; Kuroda et al., 2007; Unnanuntana et al., 2009). Collectively, these data indicate that these organisms have the capacity to interact with one another at the molecular level. Indeed, our studies demonstrated that in the *G. mellonella* model there is enhanced virulence in the dual-species infection compared to both mono-species infections, which is in agreement with previous *in vivo* murine studies also describing enhanced virulence of *C. albicans* and *S. aureus* co-infection (Carlson, 1983; Peters and Noverr, 2013). One interesting hypothesis is that *S. aureus* utilize *C. albicans* hyphae as a transportation mechanism, much like a needlestick injection, to mediate systemic infection throughout the host (Schlecht et al., 2015). This may be the reasoning behind previous findings reporting that bacteria were recovered from the kidneys of an oral candidiasis co-infection model, whereas in the mono-infection model no. *S. aureus*, were detected (Kong et al., 2015).

Given the clinical implications of the co-infection, we were then interested in evaluating miconazole as a treatment possibility, which has been shown to possess cross-kingdom efficacy (Boyen et al., 2012). Miconazole functions via a dual mechanism by primarily interfering with lipid membranes through the inhibition of ergosterol synthesis resulting in an accumulation of sterol by-products that consequently prevents cell growth (Vanden Bossche et al., 1990). Additionally, it functions in the stimulation of reactive oxygen species (ROS) promoting cell death (Kobayashi et al., 2002; Vandenbosch et al., 2010). It was shown that 10-fold greater concentrations of miconazole were required to induce an effect against the mono- and dual-species biofilms compared to planktonic combinations, and the MIC levels in dual-species were ultimately defined by *C. albicans* (40 mg/L). As this was a simplified model on plastics we also utilized a more representative 3D model to determine the viable composition (Townsend et al., 2016), where we showed significantly more viable *S. aureus* than the mono-culture model. This resistance was also translated into the *in vivo* model where reduced sensitivity was clearly demonstrated, which we propose is facilitated by the candidal-mediated protection, and in particular the ECM as has been reported elsewhere (De Brucker et al., 2015; Kong et al., 2016).

The presence of *C. albicans* ECM within the mixed-species biofilm has been shown to protect *S. aureus* from vancomycin treatment through concentrations escalating as high as 1600 mg/mL (Harriott and Noverr, 2009). A recent study has elaborated on this work, identifying fungal β-1,3-glucan as a matrix component to which *S. aureus* can encase itself within and decrease its susceptibility to vancomycin (Kong et al., 2016). The *C. albicans* ECM (β-1,3-glucans) has also been shown to increase resistance to ofloxacin within *E. coli* dual-species biofilms (De Brucker et al., 2015). Interestingly though, of all *C. albicans* ECM constituents tested, degradation of eDNA using DNase was shown to be the second most effective way of reducing ofloxacin tolerance in this dual-species model. The presence of biofilm associated eDNA has been identified and characterized in a range of bacterial and fungal pathogens including both *S. aureus* and *C. albicans* (Lister and Horswill, 2014; Rajendran et al., 2014). *S. aureus* eDNA release has been extensively characterized (Memmi et al., 2012), though eDNA release in the mixed species biofilm is less well defined. Studies between *C. albicans* and *Pseudomonas aeruginosa* revealed that secreted bacterial components are able to induce fungal cell lysis, as well as modulating fungal virulence (Gibson et al., 2009). While it is unlikely that *S. aureus* is able to secrete reciprocal components to *P. aeruginosa*, it is conceivable that as nutrients become sparse within mature biofilms *S. aureus* may scavenge off the fungal cell wall to utilize its own growth, as was reported through our metabolomic analyses (Weidj et al., 2016). When we examined the release of eDNA into the biofilm supernatant it was shown to be phase dependent, with enhanced eDNA release from the dual-species biofilms after 12 h, continuing to increase as biofilms matured, and this increased for both organisms within the biofilm. In fact, exogenous *S. aureus* genomic DNA has been shown to positively impact *C. albicans* biofilm formation (Sapaar et al., 2014).

This study demonstrates that enzymatic degradation of eDNA of a *C. albicans* biofilm negatively affects *S. aureus* adhesion, highlighting therapeutic potential. The effective disruption of *C. albicans* matrix components to enhance antimicrobial efficacy has also been demonstrated within *in vitro* mono- and dual-species biofilms (Nett et al., 2007; Rajendran et al., 2014; De Brucker et al., 2015; Kong et al., 2016). The ability to de-stabilize biofilms through degradation of eDNA make it an attractive therapeutic target, especially in polymicrobial biofilms where they can be used to sensitize co-colonizing bacteria. Further studies of these agents are required to validate their efficacy *in vivo*. The DNase Pulmozyme^®^ is currently used in combination with antibiotics for cystic fibrosis treatment, with significant reduction in the prevalence of *S. aureus* found within treated patients compared to untreated controls (Frederiksen et al., 2006).

To summarize, this manuscript reports the close association of two commonly co-isolated nosocomial pathogens, and indicates that *S. aureus* can utilize *C. albicans* biofilm architecture to its advantage. We also demonstrate that miconazole resistance within dual-species biofilms is supported by eDNA. Further exploration of this intriguing relationship between these two organisms will aid to a better understanding of their frequent co-isolation from the human host, and may provide novel avenues of possible therapeutic strategies to combat inter-kingdom biofilm infections, particularly those infections of a topical nature such as wounds, diabetic foot ulcers, and angular cheilitis.

## Author Contributions

RK, RR, JH, ET, and BS participated in study design and experimental procedures and were responsible for preparation of the manuscript. KB and SL contributed to study design and manuscript preparation. OM participated in study design and confocal microscopy imaging and analysis and manuscript preparation. WM and CW participated in study design, analysis of the data, and contributed to the manuscript. GR conceived the study, participated in study design and was responsible for producing the final manuscript. All authors have read and approved the final manuscript.

## Conflict of Interest Statement

The authors declare that the research was conducted in the absence of any commercial or financial relationships that could be construed as a potential conflict of interest.
