# Supplementary figures and images for: Candida albicans Mycofilms Support Staphylococcus aureus Colonization and Enhances Miconazole Resistance in Dual-Species Interactions
Source: Front Microbiol. 2017 Feb 23;8:258. doi: 10.3389/fmicb.2017.00258 (PMC5322193; doi:10.3389/fmicb.2017.00258)

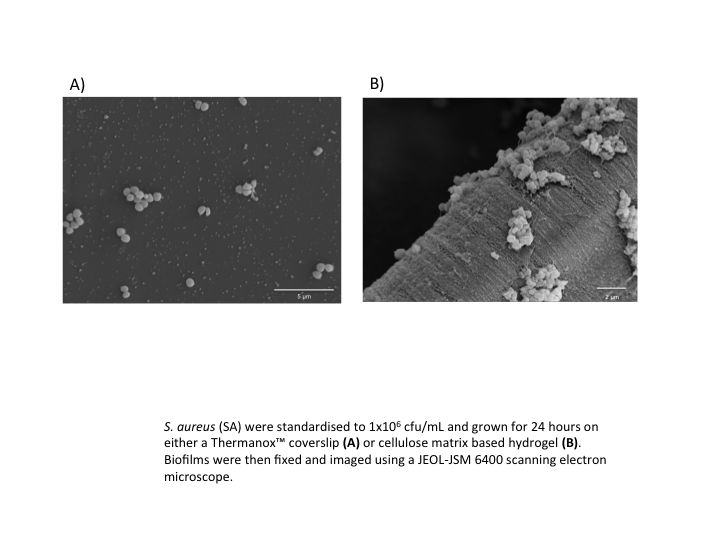

Supplement: Supplementary file 1 [file Image_1.tiff]

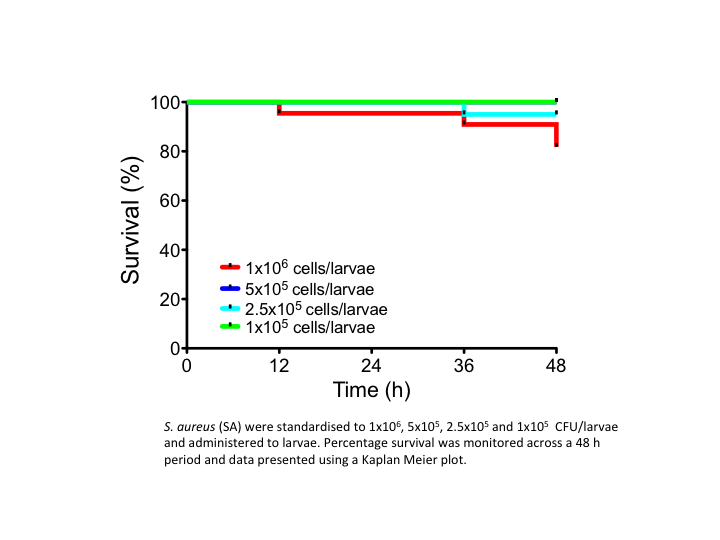

Supplement: Supplementary file 2 [file Image_2.tiff]
